# Fine-grained image classification method based on hybrid attention module

**DOI:** 10.3389/fnbot.2024.1391791

**Published:** 2024-05-03

**Authors:** Weixiang Lu, Ying Yang, Lei Yang

**Affiliations:** ^1^School of Computer, Electronics and Information, Guangxi University, Nanning, China; ^2^Guangxi Academy of Sciences, Nanning, China

**Keywords:** fine-grained image classification, spatial attention module, channel attention module, attention erasure module, ResNet50 pooling layer

## Abstract

To efficiently capture feature information in tasks of fine-grained image classification, this study introduces a new network model for fine-grained image classification, which utilizes a hybrid attention approach. The model is built upon a hybrid attention module (MA), and with the assistance of the attention erasure module (EA), it can adaptively enhance the prominent areas in the image and capture more detailed image information. Specifically, for tasks involving fine-grained image classification, this study designs an attention module capable of applying the attention mechanism to both the channel and spatial dimensions. This highlights the important regions and key feature channels in the image, allowing for the extraction of distinct local features. Furthermore, this study presents an attention erasure module (EA) that can remove significant areas in the image based on the features identified; thus, shifting focus to additional feature details within the image and improving the diversity and completeness of the features. Moreover, this study enhances the pooling layer of ResNet50 to augment the perceptual region and the capability to extract features from the network’s less deep layers. For the objective of fine-grained image classification, this study extracts a variety of features and merges them effectively to create the final feature representation. To assess the effectiveness of the proposed model, experiments were conducted on three publicly available fine-grained image classification datasets: Stanford Cars, FGVC-Aircraft, and CUB-200–2011. The method achieved classification accuracies of 92.8, 94.0, and 88.2% on these datasets, respectively. In comparison with existing approaches, the efficiency of this method has significantly improved, demonstrating higher accuracy and robustness.

## 1 Introduction

The task of fine-grained image classification refers to distinguishing different subcategories within the object class. Fine-grained image classification is a unique form of image classification task, which necessitates discrimination between semantic and instance levels, while considering the similarity and diversity among categories (Li et al., 2021). For instance, this includes identifying various breeds of dogs, and distinguishing styles of cars and airplanes. The subtle object variations within subclasses make this problem more challenging than traditional classification problems. In recent years, significant improvements in various tasks have been achieved through the use of convolutional neural networks (CNNs), such as in face recognition (Schroff et al., 2015), autonomous driving (Bojarski et al., 2016), pedestrian identification (Sun et al., 2018), and text classification. Fine-grained image classification poses a significant challenge because the categories of fine-grained images are not very different from each other, while the images within the same category can vary greatly. Traditional CNNs are not effective in extracting the subtle features of these images, leading to poor classification performance. Thus, a key issue is enabling CNNs to identify and learn the parts and features that distinguish between categories. Initial works (Zhang et al., 2014; Lin et al., 2015)relied on fixed rectangular bounding boxes and part annotations to obtain visual differences. However, acquiring additional annotation information demands extensive human effort, making these methods impractical. Therefore, researchers in recent years have paid more attention to weakly supervised fine-grained image classification tasks that only require image tags as supervision. Some approaches have developed a localization subnetwork to pinpoint critical parts, followed by a classification subnetwork for classification, such as STN (Jaderberg et al., 2015), RA-CNN (Jianlong et al., 2017), NTS-Net (Yang et al., 2018). These classification networks capture key parts of information by extracting local areas on the original image. By first identifying several discriminative local regions in the image and then cropping these regions, these models facilitate learning while maintaining high accuracy without the need for pre-selected boxes.

The efficacy of fine-grained image classification depends on the degree of variance within local regions. However, prevalent research methodologies often focus solely on identifying a single discriminative region for classification, overlooking the synergy and complementary nature of other regions. To enhance the performance of fine-grained image classification, it is imperative to integrate information from various regions rather than relying on a singular region. Hence, the objective of this paper is to identify multiple discriminative regions simultaneously and explore methods for the organic integration of information across these regions.

This paper introduces a model structure that combinates features from different levels via an attention module, thereby augmenting the semantic and discriminative capacity of features for fine-grained image classification. This framework is adept at both partial granularity learning and cross-granularity feature fusion. It comprises several key components: (1) A hybrid attention module (MA), designed to diminish noise interference within the image and enhance feature differentiation by concentrating on crucial regions; (2) An attention erasure module (EA), which identifies additional relevant features by eradicating the prominent parts of the image; (3) An enhanced ResNet50 pooling layer, which allows the model to more effectively capture global information by aggregating data from multiple regions efficiently.

The main contributions of this paper are as follows:

1. In this paper, we propose a novel fine-grained image classification model that employs a hybrid mechanism combining spatial and channel attention. This mechanism is capable of accurately locating key parts of an image and extracting highly discriminative and detailed features from these parts, thus improving the accuracy of classification;
2. We instruct the model to spread its focus by integrating an attention erasure module to avoid over-concentrating on salient features of the image. This strategy enables the model to discover and learn other key discriminative details in the image, which in turn improves the level of detail and accuracy of classification;
3. In this paper, we conduct extensive comparison and ablation experiments on three widely-adopted fine-grained image classification datasets, including CUB-200-2011, Stanford-Cars, and FGVC-Aircraft. the experimental results demonstrate the excellent classification performance of our method.

## 2 Related work

### 2.1 Fine-grained image classification

Based on the foundation of recognizing basic categories, there is also a need for more refined subcategory classification in fine-grained image classification, such as distinguishing between species of butterflies, brands of cars, and categories of fish. Categorizing images with subtle differences among subcategories presents an essential and challenging problem in computer vision, applicable in various contexts like biodiversity conservation, product retrieval, and art appreciation. The challenge stems from fine-grained images exhibiting large intra-class variance and small inter-class variance, meaning there are significant variations within the same category, while images across different categories appear very similar. Additionally, fine-grained images are affected by factors like posture, viewing angle, illumination, occlusion, and background interference, complicating classification further. Fine-grained image classification methods are broadly categorized into two types: those relying on deep learning and traditional methods based on feature extraction. Traditional feature extraction methods necessitate manually designed features, such as edge detection, color histograms, feature point matching, and visual word bags, which have limited expressive capabilities and require extensive annotation details like bounding boxes and key points. The drawback of these methods lies in the extensive manual intervention required for feature selection and extraction. Due to the impressive outcomes of deep learning, most recognition frameworks now depend on advanced convolutions for feature extraction (Krizhevsky et al., 2012). Features extracted through convolution are learned automatically by multilayer convolutional neural networks, offering the model greater adaptability to various tasks and datasets, with features possessing enhanced expressive and abstract capabilities. The benefit of convolutional feature extraction is its ability to perform feature extraction and classification within the same network, with the quality and quantity of features adjustable through the network’s structure and parameters. To augment the discriminability and diversity of convolution-extracted features, some methods introduce techniques such as attention mechanisms, contrastive learning, and logical reasoning to improve feature attention, contrast, and logic. Ding et al. (2019) proposed sparse selective sampling to learn discriminative and complementary regions, addressing the potential depletion of environmental information with locally cropped features. Traditional approaches often focus on local feature extraction, possibly neglecting environmental context. Thus, they suggest amplifying local features while preserving surrounding environmental information, using a sparse selective sampling layer for extracting multiple regions from the original image and a fusion layer for integrating these region’s features, enhancing feature completeness and diversity. Zhuang et al. (2020) recommended identifying contrastive clues through pairwise image comparisons, introducing the attention pairwise interaction network API-Net. Through pairwise interaction, contrast clues distinguishing them can be adaptively identified from a pair of detailed images. It is suggested that these contrast clues aid in enhancing the model’s ability to distinguish detailed images. Consequently, a methodology involving the utilization of two images as input, an attention module for extracting contrast clues, and an interaction module for comparing these clues is proposed to enhance feature discrimination. Specifically, a self-attention layer is employed to extract contrastive cues from the two images, while a convolutional layer is utilized to assess the similarity of these cues, thereby enhancing feature attention and contrast. Shi et al. (2020) developed a logic-based feature extraction model (LAFE), which retains discriminative features from distinguishable parts while eliminating confusing ones. LAFE utilizes regional and channel attention modules to capture distinctive and ambiguous features, introducing two novel loss functions to focus attention on these features for improved fine-grained image classification performance. Bera et al. (2022) introduced a graph neural network-based fine-grained classification model (SR-GNN), which conducts relation-aware visual feature transformation to re-allocate the significance of relevant local features and aggregate them into expressive feature representations, further advancing the field of fine-grained image classification.

### 2.2 Attention module

The attention module serves as a mechanism for neural networks to emulate human visual attention, enabling the model to concentrate on pivotal regions of an image while disregarding extraneous information during image processing. This module enhances the model’s ability to comprehend and depict the image’s significance and shape, thereby improving the expressiveness and recognition capabilities of the image. In the context of fine-grained image classification tasks, attention modules prove to be a potent tool, facilitating the automatic identification of salient image regions to serve as primary criteria for classification. This enhancement not only improves the model’s accuracy and speed in classification tasks but also reduces the model’s complexity and computational demands. Attention modules have gained widespread application across various scenarios in recent years. SENet (Hu et al., 2018), pioneering the use of a channel attention mechanism, aims to diminish the influence of less significant channels. It represents a neural network architecture that employs a channel attention mechanism to enhance the expressiveness of features. By integrating a global average pooling layer and two fully connected layers following each convolutional layer, SENet learns the significance of different channels and accordingly weights the feature maps. This design allows the model to autonomously modulate feature distribution and representation in response to various tasks and datasets. CBAM (Woo et al., 2018), a convolutional block attention module, implements an attention mechanism that assigns weights to the features across different channels and then across different locations, thereby facilitating attention across both spatial and channel dimensions to boost the model’s performance. Through the utilization of a convolutional layer and a max-pooling layer, CBAM achieves spatial attention, enhancing the model’s ability to focus on relevant areas within an image for more effective feature processing and classification.

CBAM enhances the spatial sensitivity of features through a spatial attention mechanism by incorporating a convolutional layer and a max pooling layer after each convolutional layer. This approach facilitates learning the significance of various spatial locations and weighting the feature maps accordingly. As a result, the model can autonomously adjust the spatial distribution and representation of features in alignment with different spatial scales and shapes. However, these mechanisms, focusing primarily on the visual features of local receptive fields, overlook the interconnections among global spatial channels. The concept of global spatial channels’ interrelationship involves the dependencies and influences among different spatial positions and channels, which is crucial for improving the model’s comprehension of both general and specific image features, thereby enhancing the semantics and structure of the features. Liu et al. (2021) introduced a “global” attention module aimed at reducing information loss during transmission by optimizing the algorithm and network structure, ensuring data integrity and accuracy. This module also enhances the representation of global interactions, enabling the network to better understand and process complex data relationships, which in turn improves the accuracy of its predictions and analysis. The global attention mechanism employs 3D displacement and multilayer perceptrons for channel attention and a convolutional spatial attention sub-module to heighten classification accuracy. However, this approach necessitates a global analysis of the relationship between each channel and spatial location, demanding extensive global comparisons and computations across features of each channel and location to determine attention weights. This requirement increases the network’s computational complexity and memory usage. Zhang et al. (2021) proposed a progressive joint attention network that fosters interaction among feature channels and suppresses dominant areas to divert focus to other relevant regions. This method, through the use of multiple joint attention modules, enhances the model’s capacity to use information across feature channels, enabling a more accurate representation of the data and its intrinsic qualities; thus, improving the model’s understanding and learning efficiency. Sun et al. (2018) developed a novel feature extraction module, One-Squeeze Multi-Excitation (OSME), to tackle the challenges of fine-grained image recognition. This module first produces a squeeze vector from the input feature map using global average pooling, then generates multiple excitation vectors via different excitation branches, each targeting a distinct attention region. These excitation vectors are then multiplied with the input feature map and concatenated, resulting in a composite feature vector that is both diverse and robust. In practical implementations, Sun et al. (2018) utilized two excitation branches, allowing for the extraction of two discriminative parts from the same image. Thus, enhancing the model’s effectiveness in fine-grained image recognition tasks.

## 3 Methods of this article

### 3.1 Model structure

**Figure 1:**
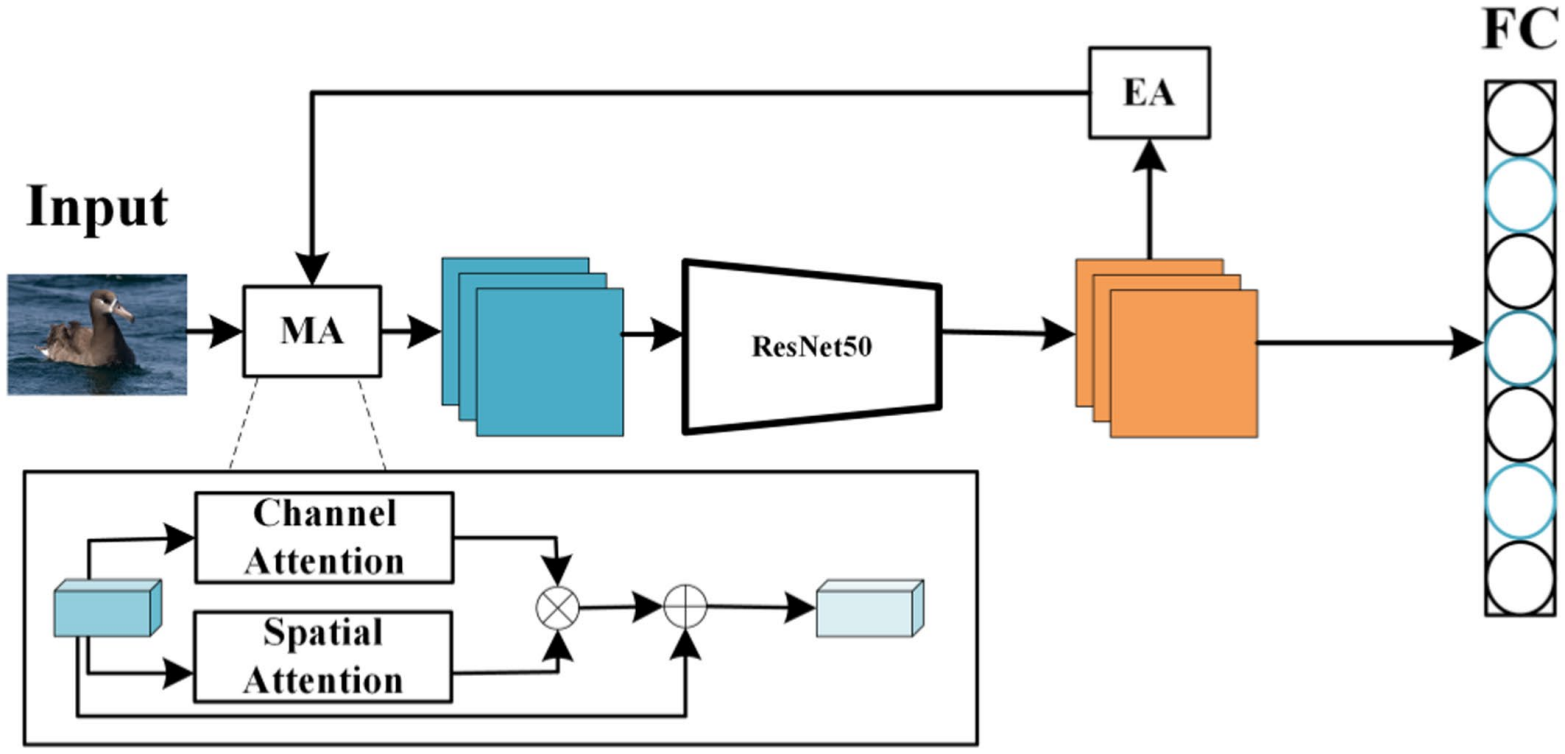
The overall structure of the network.

Given the challenge posed by the high similarity among different subcategories, it is crucial to capture the complementary information of similar areas. Traditional convolutional networks, such as ResNet (He et al., 2016), VGG (Simonyan and Zisserman, 2014), GoogleNet (Szegedy et al., 2014), exhibit good performance in general classification tasks. However, there remains significant potential for enhancement in their ability to effectively capture and utilize discriminative feature information and to efficiently filter and eliminate irrelevant information in the context of fine-grained image classification. This article introduces a fine-grained image classification method using hybrid attention, which eliminates the need for additional manual annotation and achieves superior fine-grained accuracy using only category labels. The proposed classification network, centered on hybrid attention, comprises three main components: ResNet50 serving as the feature extractor, a hybrid attention unit (MA), and an attention-erasing unit (EA). The comprehensive network structure is depicted in Figure 1.

As illustrated in Figure 1, this paper introduces an image classification model that utilizes a hybrid attention module (MA) in conjunction with an attention erasure module (EA). The core objective of this model is to employ the hybrid attention module (MA) to accentuate the prominent features within the image while eliminating irrelevant background details, followed by the extraction of high-level semantic features via the ResNet50 network. To derive a broader spectrum of features from the image, the attention erasure module (EA) is deployed, capable of automatically removing relevant areas in the image based on the features it has learned. This process enables the model to shift its focus to other, undiscovered features. Subsequently, a classifier is applied to categorize the images. This model facilitates end-to-end training without the necessity for additional annotation, achieving superior performance compared to existing methodologies across various image classification datasets.

### 3.2 Hybrid attention module

**Figure 2:**
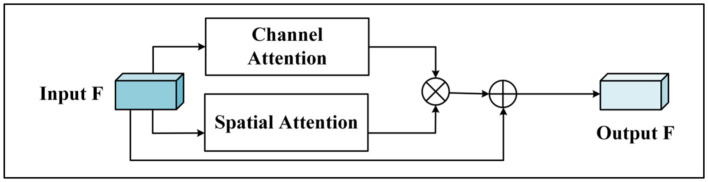
Hybrid attention module (MA).

Current attention mechanisms, including CBAM, employ a prevalent approach involving both spatial and channel attention units. CBAM orchestrates these units in a sequential manner, initially applying spatial attention followed by channel attention. This methodology facilitates the enhancement of feature representation capabilities across both spatial and channel dimensions. However, this approach exhibits a limitation wherein the feature map from the preceding module—be it the channel or spatial attention module—dictates the weights for subsequent attention modules. Consequently, the original feature map, which is pivotal for feature delineation, is overlooked. This oversight can introduce interference and diminish the classification efficacy. Additionally, traditional attention modules like SENet overlook the critical aspect of spatial significance. BAM (Park et al., 2018) attempts to integrate spatial and channel attention by mere addition, which does not effectively combine the two types of information. To address these challenges and capture a more comprehensive array of feature information, this article introduces a novel hybrid attention mechanism module, as depicted in Figure 2.

The process involves initially treating the input features through both the channel attention unit and the spatial attention unit independently, followed by the execution of subsequent operations. The weights of the channel dimension and the spatial dimensions are then mapped onto the spatial-channel feature map through matrix multiplication, culminating in the combination of the input features with the outcomes derived from the hybrid attention. The specific steps are detailed as follows:

1. Given the feature map $F\epsilon R^{C\times H\times W}$ , the channel attention map $M_{c}\epsilon R^{C\times 1\times 1}$ and the spatial attention map $M_{s}\epsilon R^{1\times H\times W}$ are derived from the channel attention module and the spatial attention module, respectively;
2. The channel-dimensional feature information is procured by conducting a multiplication of the input feature map F with the channel attention map $M_{c}$ , as delineated in Formula (1).

(1) $$F_{c}=M_{c}\left(F\right)⊗F$$

1. The spatial attention map, $M_{s}$ , applies weights to the input feature map, F , thereby acquiring the spatial dimension of the feature representation. This process is shown in Formula (2):

(2) $$F_{s}=M_{s}\left(F\right)⊗F$$

1. The product of channel attention map and spatial attention map is implemented by matrix multiplication so that the model can learn specific feature information, as shown in Formula (3):

(3) $$F^{\prime }=F_{c}⊗F_{s}$$

1. The final feature representation is derived by summing the original input feature map with the output from the hybrid attention module. This is expressed as Formula (4):

(4) $$F_{\mathrm{out}}=F^{\prime }+F$$

1. The process can be expressed by the Formula (5):

(5) $$F_{\mathrm{out}}=F+\left(F⊗M_{s}\left(F\right)⊗M_{c}\left(F\right)\right)$$

#### 3.2.1 The spatial attention module

The spatial attention module is designed to minimize background distractions. To focus the network on essential information within the image and eliminate irrelevant data, this module incorporates dilated convolution and employs a pyramid scheme to expand the receptive field without sacrificing detail. Such measures significantly enhance classification accuracy.

**Figure 3:**
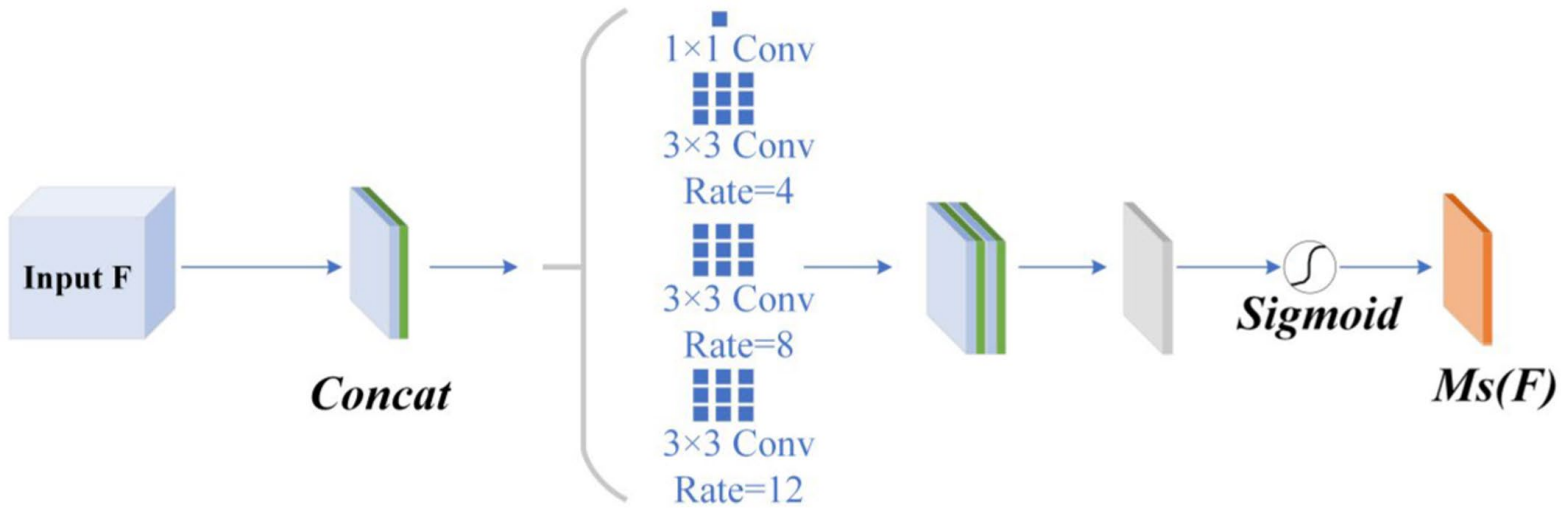
Spatial attention structure.

The input feature map $F\epsilon R^{C\times H\times W}$ is processed by the spatial attention module, which enhances the features in the spatial aspect. The spatial attention module first performs average pooling and max pooling across the feature map F channel dimension, merges the resulting 2×H×W feature maps into a single 2×H×W map, and then applies a convolutional layer to extract features. A 7×7 convolutional layer followed by a sigmoid activation function generates a H×W spatial attention weight. The convolutional and pooling layers are pivotal to the spatial attention module’s functionality. Figure 3 displays the detailed architecture.

Specific process: Initially, the channel information of the input feature map is acquired through the application of global maximum pooling and global average pooling. At this time, two feature maps are obtained: $F_{avg}^{s}\epsilon R^{1\times H\times W}$ , $F_{\text{max}}^{s}\epsilon R^{1\times H\times W}$ , as shown in the Formulas (6, 7):

(6) $$F_{avg}^{s}=\mathrm{Avgpool}\left(F\right)$$

(7) $$F_{\text{max}}^{s}=\mathrm{Maxpool}\left(F\right)$$

The two feature maps are spliced together to obtain the maximum pooling and average pooling feature maps $F_{\mathrm{concat}}\in R^{2\times H\times W}$ , as shown in the Formula (8):

(8) $$F_{\mathrm{concat}}=\mathrm{Concat}\left(F_{\text{max}}^{s},F_{avg}^{s}\right)$$

The Atrous Spatial Pyramid Pooling (ASPP) module, which includes four branches, is introduced. This module comprises a 1×1 conventional convolution layer and three 3×3 atrous convolution layers with dilation rates of 4 and 8, respectively. Each branch undergoes normalization and incorporates rectified linear unit. Through this process, multi-scale information from the feature map is captured, maintaining uniform channel numbers. After processing by the atrous convolution module, the outputs from the four branches are unified to dimensions of 1×H×W. Subsequently, the extracted feature information from these branches is concatenated, resulting in $F^{’}$ belonging to $F^{’}\in R^{4\times H\times W}$ , as indicated in the following Formula (9):

(9) $$F^{’}=C\mathrm{oncat}\left(\mathrm{ASPP}\left(F_{\mathrm{concat}}\right)\right)$$

The concatenation approach multiplies the number of channels by four, prompting this study to revert to the original channel count via a convolution layer with a single channel and a 7 × 7 kernel size. Subsequently, the feature map is outputted following batch normalization and processing through a rectified linear unit. Concurrently, the Sigmoid activation function is employed, ensuring that the range of each weight is between 0 and 1, as illustrated in the Formula (10):

(10) $$F^{\prime \prime }=\mathrm{Sigmoid}\left(\mathrm{Conv}7\times 7\left(F^{\prime }\right)\right)$$

The overall formula of the spatial attention module is as Formula (11):

(11) $$M_{s}\left(F\right)=\mathrm{Sigmoid}\left(\mathrm{Conv}7\times 7\left(\mathrm{Concat}\left(\mathrm{ASPP}\left(\begin{array}{l} \mathrm{Avgpool}\\ \left(F\right);\\ \mathrm{Maxpool}\\ \left(F\right) \end{array}\right)\right)\right)\right)$$

Among them, $M_{s}\left(F\right)\epsilon R^{1\times H\times W}$ , represents the final output spatial weight matrix.

#### 3.2.2 The channel attention module

**Figure 4:**
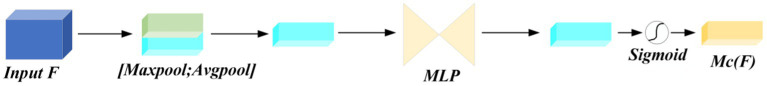
Channel attention structure.

This study aims to enhance the distinctiveness and expressiveness of features by using the interaction among feature map channels. Traditionally, attention mechanisms have relied on either average or max pooling to reduce spatial dimensions. However, this paper posits that maximum and average pooling can highlight different aspects of feature information, and thus, employs both pooling methods simultaneously. Unlike the conventional hybrid attention model CBAM, which merely combines the outcomes of the two pooling approaches, this paper argues that such a method does not adequately integrate feature information. To better capture and merge both global and local key information, the paper initially merges the results of maximum and average pooling. Subsequently, it modifies the number of channels via a convolution layer. The specific architecture is depicted in Figure 4.

The process is outlined as follows: The input feature map FϵRC×H × W, is subjected to both max pooling and average pooling operations. This results in the creation of two feature maps along the channel dimension: $F_{avg}^{c}\epsilon R^{C\times 1\times 1}$ and $F_{\text{max}}^{c}\epsilon R^{C\times 1\times 1}$ , as demonstrated in the Formulas (12, 13):

(12) $$F_{avg}^{c}=\mathrm{Avgpool}\left(F\right)$$

(13) $$F_{\text{max}}^{c}=\mathrm{Maxpool}\left(F\right)$$

Within this context, average pooling and maximum pooling are represented by average pooling and maximum pooling, respectively.

$F_{\mathrm{concat}}\epsilon R^{2C\times 1\times 1}$ is obtained by fusing the two pooling results, as shown in the Formula (14):

(14) $$F_{\mathrm{concat}}=\mathrm{Concat}\left(F_{avg}^{c},F_{\text{max}}^{c}\right)$$

To reduce the channel count from 2C to C for the concatenated feature map, a 1 × 1 convolutional layer is utilized to compress the channels of the concatenated feature map. This is expressed as Formula (15):

(15) $$F^{\prime }=Conv1\times 1\left(F_{\mathrm{concat}}\right)$$

The feature map F′ is input into the MLP network, which performs dimensionality reduction. Specifically, the channel attention map $M_{c}\epsilon R^{C\times 1\times 1}$ is produced through two fully connected layers. The channel attention weight is then derived by applying the Sigmoid function as an activation function to the feature map. Subsequently, the output of the channel attention module is generated by multiplying the feature map by the corresponding weight, as illustrated in the Formula (16):

(16) $$M_{c}=\mathrm{Sigmoid}\left(MLP\left(F^{\prime }\right)\right)$$

The Formula (17) is the general equation for channel attention:

(17) $$M_{c}\left(F\right)=\mathrm{Sigmoid}\left(MLP\left(\mathrm{Conv}\left(\mathrm{Concat}\left(\begin{array}{l} \mathrm{MaxPool}\left(F\right),\\ \mathrm{Avgpool}\left(F\right) \end{array}\right)\right)\right)\right)$$

### 3.3 Attention erasure module (EA)

In image classification tasks, the attention module serves as a potent mechanism for boosting model efficiency by allocating greater significance to relevant information in the image. Nonetheless, this module may also present limitations, potentially causing the model to overlook fine details in the image, thereby diminishing its generalization capability. The intricacy of fine-grained image classification lies in the subtle distinctions between subcategories, which are often challenging to discern, necessitating the consideration of various image features for precise classification. To mitigate this challenge, this article introduces the attention erasure (EA) module, designed to automatically excise regions identified by the attention module, thereby redirecting the model’s focus toward previously overlooked areas within the image. This approach enables the model to uncover additional image features, consequently enhancing classification accuracy.

The process initiates with average pooling applied to the feature map generated by the attention module, followed by the employment of an up-sampling technique to adjust it to the size of the original image, as shown in the Formula (18):

(18) $$A\left(H,W\right)=\mathrm{Upsample}\left(F^{\prime }\left(H,W\right)\right)$$

Then, a threshold θ is set, and the values above θ are set to 0, while the others are set to 1, and the mask M (H, W) is obtained as shown in the Formula (19):

(19) $$M\left(H,W\right)=\{\begin{array}{c} 0,A\left(H,W\right)>\theta \\ 1,\mathrm{else} \end{array}$$

Cover the mask $M\left(H,W\right)$ on the attention map to get the new deleted area image, as shown in the Formula (20):

(20) $$F^{\prime \prime }=F^{\prime }⊗M$$

By selectively obscuring regions within the image, the neural network can be made to not only focus on salient features but also to derive additional information across the entire image. Such a strategy can augment the network’s capacity for generalization, thereby maintaining consistent performance across diverse training samples.

### 3.4 Improved ResNet50 pooling layer

**Figure 5:**
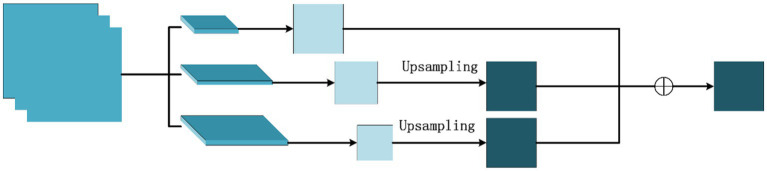
Improved pooling layer structure.

This study integrates the concept of the pyramid pooling module and proposes an advanced ResNet50 pooling layer to enhance the perceptive field and feature extraction capabilities of the network’s initial layers. Following the primary convolutional layer in ResNet50, a 3×3 max pooling layer is employed to reduce both the image’s resolution and noise levels. However, this reduction may lead to a loss of certain image details and structural information, negatively impacting the network’s efficiency. To address this issue, the conventional max pooling layer is substituted with three maximum pooling layers of varying scales: 2×2 , 4×4 , and 8×8 . These layers, corresponding to different receptive field sizes, are capable of capturing varied levels of image features. To maintain the image’s resolution without diminishing it, an up-sampling procedure--bilinear interpolation is applied to the outputs of the 4×4 and 8×8 max pooling layers, ensuring that their output feature maps align with those produced by the 2×2 max pooling layer in terms of characteristics. Subsequently, the three feature maps are combined through element-wise addition, yielding a composite and potent feature map that proceeds to the subsequent convolutional layer. The architecture of the enhanced pooling layer is depicted in Figure 5.

### 3.5 Loss function

In this article, cross-entropy is employed as the loss function for optimizing model parameters. Predictions at each stage are based on information of different granularity, making them unique and complementary. The integration of outputs from all stages, with equal weighting, is anticipated to improve the model’s performance. Thus, the total classification loss presented is the sum of the classification losses from each stage. In the first stage, the input image is weighted by the hybrid attention module (MA), then feature extraction and classification are performed using the ResNet50 network. In the second stage, the attention erasure module (EA) removes the prominent regions identified in the attention map from the first stage, shifts focus to other regions, and the ResNet50 network is employed again for feature extraction and classification. Throughout this process, the loss values from both stages are cumulatively calculated, as shown in the Formula (21):

(21) $$Loss\left(y^{p},y\right)=-\overset{2}{\underset{i=1}{\sum }}y_{i}^{p}*\log\left(y_{i}^{p}\right)$$

Among them, $y^{p}$ is the predicted value.

## 4 Experimental results

### 4.1 Data sources and evaluation measures

Addressing the intricate issue of fine-grained image classification, this paper has chosen three extensively utilized datasets for experimental validation, which include Stanford Cars (comprising 196 car image categories), FGVC-Aircraft (including 100 aircraft image categories), and CUB-200–2011 (incorporating 200 bird image categories), as depicted in Table 1.

**Table 1:** Statistical characteristics of the data set.

Data set	Number of categories	Number of training set samples	Number of test set samples
Stanford Cars (Krause et al., 2013) FGVC-Aircraft (Maji et al., 2013) CUB-200-2011 (Wah et al., 2011)	196 100 200	8,144 6,667 594	8,041 3,333 5,794

The evaluation metric for the classification approach proposed in this study is classification accuracy, denoted as Accuracy. The accuracy is calculated as in Formula (22):

(22) $$A\mathrm{ccuracy}=\frac{T_{n}}{A_{n}}\times 100\%$$

The test set accuracy is denoted by $T_{n}$ and $A_{n}$ , where $T_{n}$ represents the count of samples correctly predicted, and $A_{n}$ signifies the total sample count within the test set.

### 4.2 Experimental details

The experiments detailed in this paper were performed on a server equipped with an RTX 3090 GPU (24GB), featuring 48 CPU cores and 80GB of RAM. Training relied solely on the image class label for annotations. Input images were resized to 550 × 550, then randomly cropped to 448 × 448 for model training, with data augmentation techniques applied. During testing, input images were resized to 550 × 550 and centrally cropped to 448 × 448. The optimizer utilized in this research is SGD, configured with a momentum of 0.9, a weight decay of 0.0006, and a batch size of 16. The initial learning rate was set at 0.002.

### 4.3 Comparative test

#### 4.3.1 Introduction to comparison methods

B-CNN: this method employs two separated neural networks to extract image features separately. Based on this, it employs a bilinear pooling technique to perform an outer product operation on these two sets of feature tensors, thus effectively revealing information about the interactions between these features.

BAM B-CNN: the method uses a bilinear convolutional neural network fine-grained image classification algorithm based on an attention mechanism to obtain an activation mapping map of the original image via a VGG-16 network and extract candidate regions using a region proposal network (RPN). A channel attention module is also introduced to learn the nonlinear relationship between channels to improve the expressiveness of key features.

DAG-CNN: the method fuses multiple scales of spatial potential representations from different layers of the residual network. The attention network is then used to propose and filter key sections based on the attention graph and select differentiated key regions by filtering them with channel attention weights optimising the importance of sections at different scales.

CSE: the method fully extracts the salient features in the target by focusing on the most salient features and suppressing some of the sub-salient features. By fusing these features, an efficient fine-grained feature representation can be obtained.

SE-HBP: initially, the method creates a saliency map through a dedicated network to emphasize the important areas within the image. Subsequently, a deep convolutional neural network extracts features, which are then integrated with the saliency map. This integration is enhanced by hierarchical bilinear pooling, ensuring a detailed and structured representation of the images features. The culmination of this process involves using the enriched features to train a classifier, aimed at precise image classification tasks.

MBP: the method employs a fine-grained visual classification approach that combines multilayer bilinear pooling with object localization. This method uses an object localization module to identify key objects in the image and extracts features through multilayer bilinear pooling, while suppressing background noise, thus obtaining a more refined feature representation.

#### 4.3.2 Analysis and discussion of results

The methodology of this study was compared with other state-of-the-art techniques on the CUB-200-2011, Stanford Cars and FGVC-Aircraft datasets, and the relevant results are presented in Table 2. The table shows the base model and the accuracy of each model in fine-grained image classification tasks.

1. In the evaluation of the CUB-200-2011 dataset, the methods used in this study performed well with an accuracy of 88.2%, which is significantly higher than the other compared methods. These methods usually focus only on the most salient features in an image and ignore background information as much as possible. In contrast, the method in this study not only captures the main features, but also effectively identifies other key features in the image through the application of the attentional erasure module, a strategy that greatly improves the classification accuracy.
2. In the FCVC-Aircraft dataset, the method in this paper achieves 94.0% accuracy, outperforming other methods. The CSE method starts from channel information, captures salient features in different channels of the original image, and ensures the complementarity between these features. The idea of this study is similar to CSE but different. The method in this paper not only pays attention to the channel information, but also to the features of spatial information, and enhances the classification by the complementary information of these two dimensions. The experimental results on the FCVC-Aircraft dataset proved that the accuracy of this study’s method is higher, thus validating the superiority of its method.
3. As can be seen from Table 2, the method in this paper obtains 92.8% accuracy with ResNet50 as the base model, which is better than the other methods. The method described in this paper, through the MA module and EA module, as well as the improved pooling layer, accurately focuses on the position of local regions, enhancing the network’s feature representation and the model’s generalization ability. As a result, this method demonstrates superior performance.

**Table 2:** Classification accuracy of this article’s model and other models.

Method	Basic network	CUB-200-2011	FCVC-Aircraft	Standford Cars
B-CNN (Lin et al., 2015)	VGG16	84.1	84.1	91.3
BAM B-CNN (Li et al., 2021)	VGG16	86.0	89.1	92.1
DAG-CNN (Liu et al., 2021)	ResNet50	86.2	/	/
CSE (Zhao et al., 2021)	ResNet50	87.9	92.4	93.9
SE-HBP (Chen and Chen, 2021)	ResNet34	86.5	90.8	92.9
MBP (Li et al., 2021)	ResNet34	87.7	91.1	93.8
Ours	ResNet50	88.2	92.8	94.0

### 4.4 Ablation experiment

**Figure 6:**
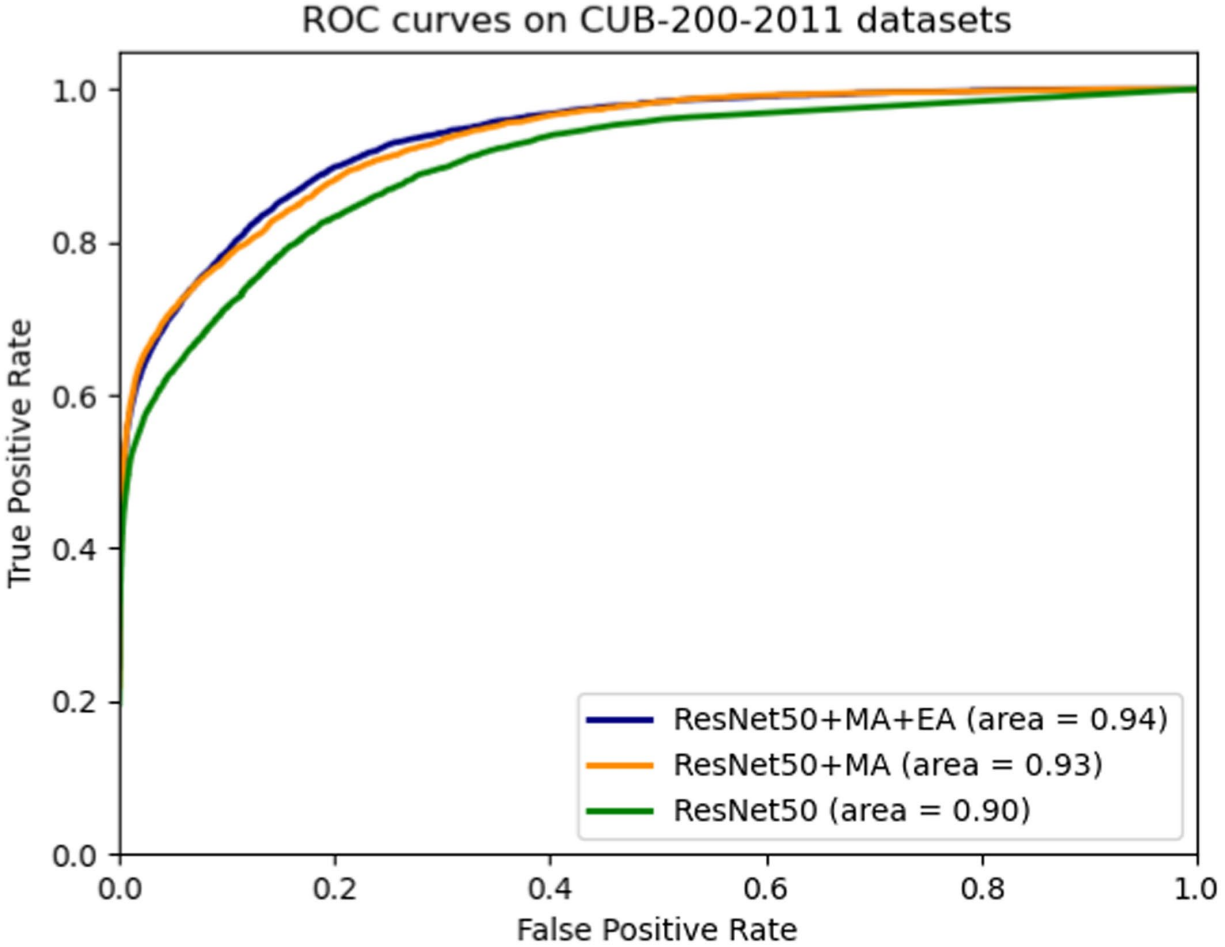
ROC curves on CUB-200-2011 datasets.

By incorporating the hybrid attention module (MA) and the attention erasure module (EA) into the ResNet50 network and enhancing the original network’s pooling layer, ablation study results for each module were obtained, as displayed in Table 3 and Figure 6. The hybrid attention module (MA) aims to enhance the feature maps’ expressiveness across various dimensions, enabling better capture of meaningful image information. The experiments demonstrated that enhancing the ResNet50 pooling layer and integrating the MA module increased model accuracy on the test set from 85.6% (original model) to 87.9%, and the AUC value also from 0.90 rose to 0.93. The attention erasure module (EA) is designed to redirect the network’s focus toward other parts of the image, thereby enhancing the model’s perceptual capabilities. The introduction of the EA module further improved accuracy to 88.2% and the AUC value also improved by 0.1. These findings indicate that the MA and EA modules can improve detail localization within images, generate more effective feature maps, and assist the network model in enhancing the accuracy of fine-grained classification.

**Table 3:** This method’s ablation experimental results on the CUB-200-2011 data set.

ResNet50	Improved ResNet50 pooling layer	MA	EA	Accuracy
√				85.6%
√	√			85.8%
√	√	√		87.9%
√	√	√	√	88.2%

### 4.5 Model complexity analysis

In this section, we use FLOPs and inference time to measure the model’s time complexity, while the parameter count is used to describe the model’s space complexity. FLOPs represent the number of floating-point operations, an indicator that reflects the computational load of the model. Inference time refers to the time required for the model to classify a single image. The parameter count, on the other hand, is the sum of parameters across all layers of the model, which is used to assess the storage space occupied by the model. As shown in Table 4, the method proposed in this study has achieved a significant improvement in model accuracy with only a minimal increase in computational and parameter requirements.

**Table 4:** Model complexity.

Model	FLOPs 10^9^ times)	Inference time(ms)	Parameter count(Mb)
ResNet50	16.5	26.4	22.4
ResNet50 + MA	18.5	33.1	24.6
ResNet50 + MA + EA	19.8	35.6	25.8

## 5 Visualizations

**Figure 7:**
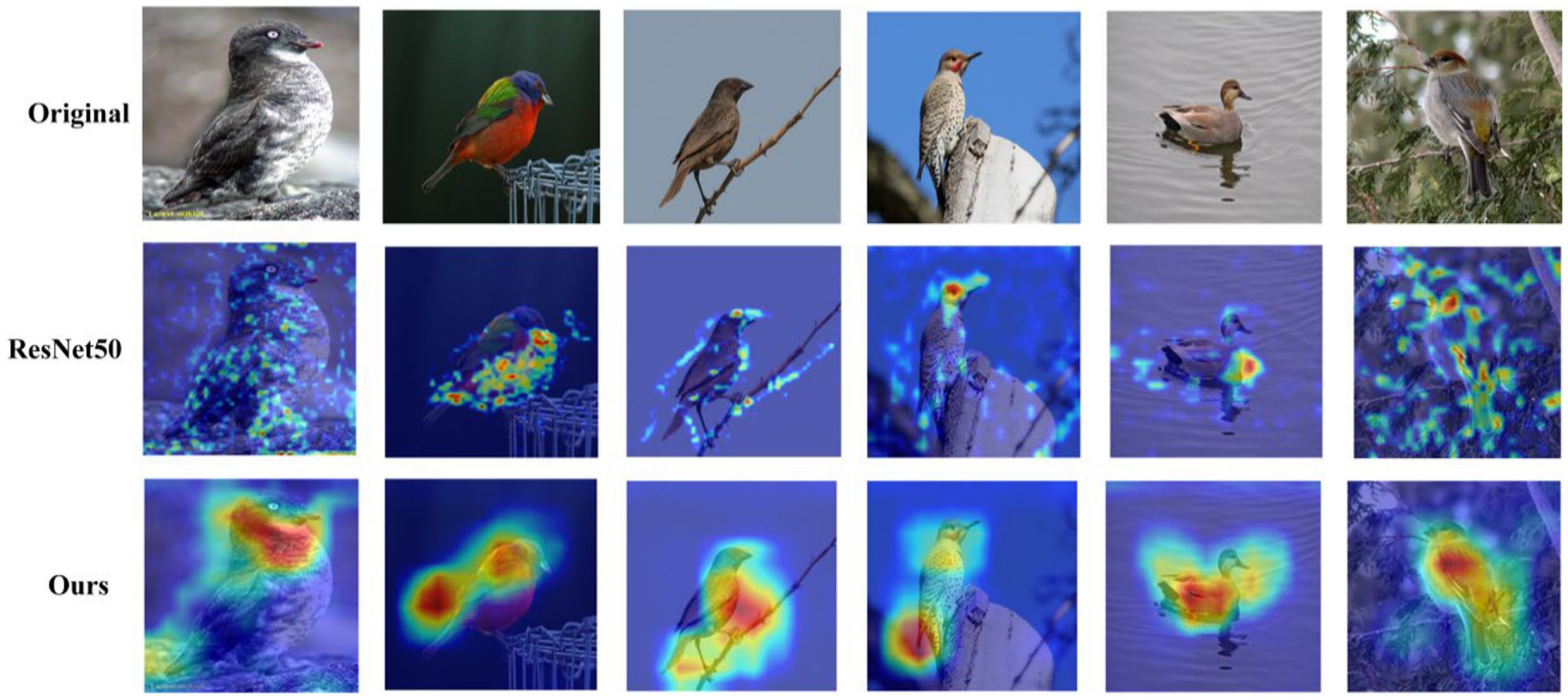
The comparative feature extraction results: the first row displays the original images, the second row shows features extracted by the ResNet50 model, and the third row depicts features extracted by our proposed method. This comparison highlights our method’s capacity to learn richer and more nuanced feature information than the basic model.

To evaluate our method’s efficacy in image classification tasks, we applied the Grad-CAM technique for visualization on the CUB-200–2011 dataset. Grad-CAM, a gradient-based visualization tool, generates heatmaps from the weighted sum of feature maps, underscoring the image regions instrumental in the classification outcome. Figure 7 presents a visual comparison between our method and the baseline model (ResNet-50), revealing that while the baseline model primarily focuses on the most conspicuous parts of the image, such as the bird’s wings, our method is capable of discerning more intricate features vital for differentiating various bird species. This underscores our method’s ability to allocate appropriate attention weights to each region, thereby rendering the classification predictions more comprehensive and precise. Furthermore, our method excels in identifying not only the prominent features but also the subtle, fine-grained characteristics essential for distinguishing between different bird types.

## 6 Conclusion

This paper introduces a novel network structure for fine-grained image classification, featuring three innovative modules: (1) Hybrid attention module (MA), which uses both spatial and channel attention to adaptively identify significant image regions; thus, augmenting the representation of global features. Spatial attention emphasizes the salient image parts, whereas channel attention modifies the weights of various feature channels, directing the network’s focus toward more distinctive features. (2) Attention erasure module (EA), which builds upon the hybrid attention module by implementing an attention erasure strategy to progressively remove previously noted image regions, encouraging the network to observe finer details. (3) Enhanced pooling layer, which upgrades the ResNet50’s pooling layer to accommodate images of varying sizes. Through extensive experimentation across multiple fine-grained image classification datasets, the effectiveness and superiority of this methodology have been confirmed. The empirical results demonstrate that this approach outperforms existing methods in classification accuracy, validating its potential in advancing fine-grained image classification research.

## Data availability statement

The raw data supporting the conclusions of this article will be made available by the authors, without undue reservation.

## Author contributions

WL: Methodology, Visualization, Writing – original draft. YY: Funding acquisition, Supervision, Writing – review & editing. LY: Funding acquisition, Supervision, Validation, Writing – review & editing.
